# Glutamate carboxypeptidase II gene knockout attenuates oxidative stress and cortical apoptosis after traumatic brain injury

**DOI:** 10.1186/s12868-016-0251-1

**Published:** 2016-04-18

**Authors:** Yang Cao, Yang Gao, Siyi Xu, Jingang Bao, Yingying Lin, Xingguang Luo, Yong Wang, Qizhong Luo, Jiyao Jiang, Joseph H. Neale, Chunlong Zhong

**Affiliations:** Department of Neurosurgery, Ren Ji Hospital, School of Medicine, Shanghai Jiao Tong University, 160 Pujian Road, Shanghai, 200127 China; Department of Psychiatry, Yale University, School of Medicine, West Haven, CT 06516 USA; Department of Biology, Georgetown University, Washington, DC 20075 USA

**Keywords:** NAAG, Glutamate carboxypeptidase II, Gene knockout, Traumatic brain injury, Oxidative stress, Apoptosis

## Abstract

**Background:**

Glutamate carboxypeptidase II (GCPII) inactivates the peptide co-transmitter *N*-acetylaspartylglutamate following synaptic release. Inhibition of GCPII elevates extracellular levels of the peptide, inhibits glutamate release and is neuroprotective in an animal model of traumatic brain injury. GCPII gene knockout mice were used to examine the cellular mechanisms underlying the neuroprotective efficacy of this transmitter system.

**Results:**

Following controlled cortical impact injury, GCPII knockout (KO) mice exhibited reduced TUNEL-positive nuclei in the contusion margin of the cerebral cortex relative to wild type mice. Impact injury reduced glutathione levels and superoxide dismutase and glutathione peroxidase activities and increased malondialdehyde. Each of these effects was moderated in KO mice relative to wild type. Similarly, the injury-induced increases in cleaved caspase-3, cytosolic cytochrome c levels and Bcl-2/Bax ratio observed in wild type mice were attenuated in the knockout mice.

**Conclusions:**

These data support the hypothesis that the neuroprotective efficacy of GCPII KO in traumatic brain injury is mediated via a reduction in oxidative stress.

## Background

Traumatic brain injury (TBI) results in immediate irreversible primary brain tissue damage and secondary delayed loss of neurons and glia in the penumbra surrounding the injury site leading to progressive emotional, cognitive and behavioral impairment [1]. Prevention of the secondary brain injury represents the main target of TBI therapy. Excessive release of glutamate following TBI and subsequent intracellular calcium (Ca^2+^) overload significantly contributes to second brain injury, including brain edema, cerebral ischemia, energy failure, mitochondrial dysfunction, abnormal oxidative phosphorylation, and neuronal death [2]. Notably, glutamate accumulation inhibits cystine uptake and leads to depletion of GSH, a crucial constituent of the anti-oxidative system [3]. Elevated intracellular Ca^2+^ makes neurons more dependent on cystine uptake for GSH synthesis, thus exacerbates the imbalanced redox status. Neuronal degeneration following excess glutamate exposure also has been attributed to oxidative damage generated in mitochondria [4]. Mitochondrial dysfunction induced by TBI causes overproduction of reactive oxygen species, depletion of adenosine triphosphate, and more importantly, leakage of cytochrome c, which is a key mediator in prompting caspase-dependent cell apoptosis [5]. As a result, inhibition of glutamate release following TBI is likely to attenuate oxidative stress and delayed cell death.

*N*-Acetylaspartylglutamate (NAAG) is one of the most abundant transmitters in the mammalian nervous system [6], and serves as a potent agonist at the type 3 metabotropic glutamate receptor (mGluR3) [7–9]. Activating mGluR3 by NAAG reduces the synaptic glutamate release [10–12]. Furthermore, elevated concentration of NAAG promotes glutamate uptake in astrocytes as a consequence of mGluR3 activation [13, 14]. Inhibition of glutamate carboxypeptidase II (GCPII) increased NAAG and reduced glutamate, aspartate, and GABA levels in a fluid percussion injury rat model [15]. In the same TBI model system, NAAG peptidase inhibition, reduced acute neuronal degeneration and astrocyte damage in the CA2/3 regions of the hippocampus [16].

These data suggest that inhibition of GCPII is a potentially significant therapeutic strategy for reducing glutamate induced secondary neurodegeneration following traumatic brain injury. However, the cellular mechanisms underlying this neuroprotection have not been established. In the present study, a GCPII knockout (KO) mouse strain has been used to assess the potential role of oxidative stress related pathways in the neuroprotective activity of NAAG. We previously reported that these GCPII KO mice develop normally and exhibit less neurodegeneration and astrocyte damage following TBI [17].

## Methods

### Animals

Male GCPII KO mice and their littermates (from the Shanghai Research Center for Model Organisms, China) in ages 8–12 weeks, weighing 25–28 g were used in this study. Genotypes of KO mice and their wild-type (WT) littermates were confirmed by PCR amplification as described in a previous study [17]. Animals were free access to food and water and housed under a 12-h light/dark cycle in a constant temperature (24 °C) and humidity (50 %) environment.

### Production of TBI

A controlled cortical impact (CCI) device (PinPoint™ PCI3000, Hatteras Instruments Inc., USA) was used to induce TBI as described previously [17]. Mice were anesthetized (sodium pentobarbital, 65 mg/kg intraperitoneally) and fixed in a stereotaxic frame. The core body temperature was monitored by a rectal probe and maintained at 37.0 ± 0.3 °C using a heating pad. A 5-mm diameter craniotomy centered at 2.0 mm posterior to bregma and 2.0 mm lateral to the midline over the right parietal cortex was performed, and injury to dura was avoided. Injury was induced by a 3.0 mm rounded metal tip that was angled to have a vertical direction to the brain surface. The metal tip compressed the brain at a speed of 3.0 m/s with a deformation depth of 1.0 mm below the dura. Apart from CCI injury, the sham group mice underwent the identical procedure as injured mice.

### Tissue collecting and sectioning

Animals (n = 6 per group) were euthanized with sodium pentobarbital (65 mg/kg intraperitoneally) at 24 h post-injury, and transcardially perfused with phosphate-buffered saline (PBS) followed by 50 ml of 4 % paraformaldehyde (PFA). The intact brains were post-fixed in 4 % PFA at 4 °C overnight and then transferred to PBS containing 30 % sucrose for cytoprotection. Consecutive coronal sections of 5 μm thickness were cut at 200 μm intervals in a vibratome (Leica VT 1000S). Every section starting at approximately bregma-1 mm and ending at bregma-3.5 mm was collected in 24-well cell culture plate for staining. A total number of twelve sections in each brain were collected for terminal deoxynucleotidyltransferase-mediated dUTP nick 30′-end labeling (TUNEL) staining.

### TUNEL staining and quantification

TUNEL staining data were obtained using a commercial kit (In situ Cell Death Detection Kit; Roche Molecular Biochemicals, Mannheim, Germany). Briefly, sections were incubated in TUNEL reaction mixture containing terminal deoxynucleotidyltransferase (TDT) for 60 min at 37 °C. The slides were then washed in PBS for three times, counterstained with 4′,6-diamidino-2-phenylindole (DAPI) for 2 min, and rinsed with PBS. The sections were analyzed under a fluorescent microscope (20× objective, Nikon 90i, Tokyo). A cell was considered as apoptotic only if its nucleus was stained with both fluorescein and DAPI. Six randomly selected non-overlapped vision fields (at 200× magnification) surrounding the primary contused area in each section (n = 6 per group) were chosen, and the mean number of TUNEL-positive nuclei/DAPI-stained nuclei in the six views was regarded as the apoptotic index of each section. The final average percentage of TUNEL-positive cells of the twelve sections was regarded as the data for each animal.

### Measurement of glutathione (GSH), malondialdehyde (MDA) levels, and superoxide dismutase (SOD) and glutathione peroxidase (GPx) activities

To detect oxidative damage and evaluate the neuroprotective ability of GCP II KO against TBI-induced oxidative injury, the entire ipsilateral cortex (n = 6 per group) was collected 24 h following TBI for analysis of the brain concentration of MDA and GSH and the activity of GPx and SOD using commercially available assay kits (Nanjing Jiancheng Bioengineering Institute, Nanjing, China).

### Protein extraction and western blot analysis

Mice were sacrificed and decapitated 1 day after CCI, and the brains (n = 6 per group) were quickly removed. A 5-mm punch of the ipsilateral cortex including 3 mm primary impacted tissue and 2 mm penumbra tissue in each brain was obtained for western blot analysis (n = 6 per group). Protein extraction of both cytosolic and mitochondrial fractions was performed as previously described [18]. Samples were homogenized in prechilled extraction buffer (Cytosol/Mitochondria Fractionation kit; Merck, Rockland, Massachusetts, USA) with a protease inhibitor and DTT, and then sedimented at 700 g for 10 min at 4 °C. The supernatant was further sedimented at 10,000 g for 30 min at 4 °C. Supernatant of 10,000 g was collected as cytosolic fraction while the pellet was resuspended with mitochondrial extraction buffer mix and saved as mitochondrial fraction. Protein concentrations were determined using the Pierce BCA kit. Equal amounts (40 μg) of proteins were separated by 12 % sodium dodecyl sulfate–polyacrylamide gels and transferred to polyvinylidene fluoride (PVDF) membranes. The membranes were blocked with 5 % skimmed milk in PBS containing 0.05 % Tween 20 (PBST) for 2 h at room temperature and then probed with primary antibodies at 4 °C overnight on a shaking platform. The primary rabbit-anti-mouse antibodies, including cleaved caspase-3, cytochrome c, Bcl-2, Bax, *β*-actin, and VDAC were 1:1000 dilutions purchased from Cell Signaling Technology, Danvers, MA, USA. The membranes were washed by PBST and incubated with goat anti-rabbit peroxidase-conjugated secondary antibody (1:5000; Bioworld Technology, Minneapolis, MN, USA) at 4 °C for 1.5 h. The relative intensity of protein signals was normalized to the corresponding *β*-actin or VDAC intensity and was analyzed by Image J software.

### Statistical analysis

All data were presented as mean ± standard error of the mean (SEM). Data were analyzed by one-way ANOVA and post hoc Bonferroni *t* test. Statistical difference was set at *p* < 0.05.

## Results

### GCPII KO attenuates oxidative stress after traumatic brain impact

**Fig. 1:**
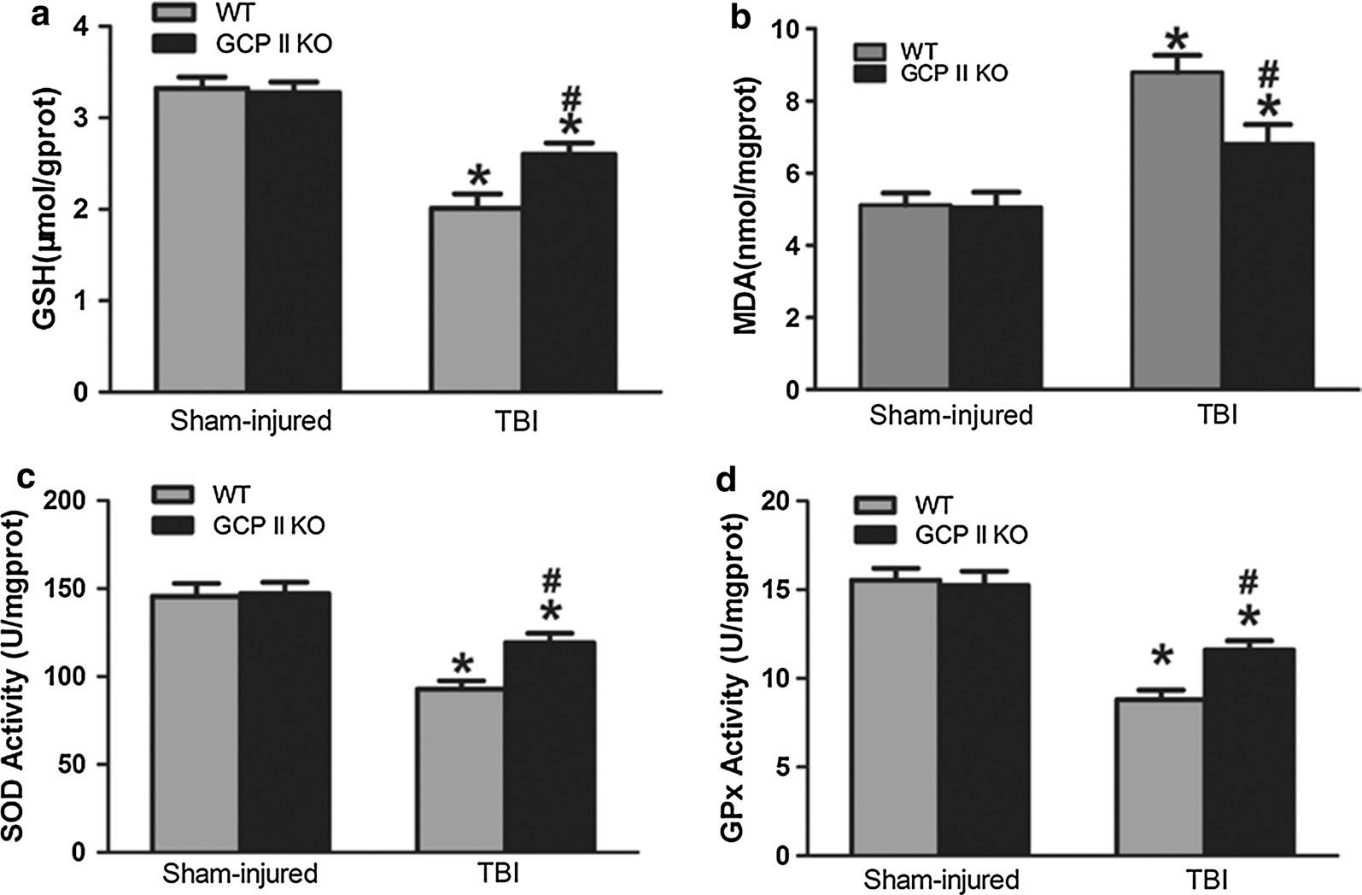
Effects of GCP II KO on GSH (**a**), MDA (**b**), SOD (**c**), GPx (**d**). TBI caused significant decreases of GSH level, SOD, GPx activities, and an increase in the MDA level. GCP II KO significantly moderated these changes while not altering the levels in sham controls. Data were represented as mean ± SEM (n = 6 per group); *p < 0.05, versus sham control of the same genotype; ^#^ *p* < 0.05, versus injured WT mice

Decreased SOD and GPx activities, GSH level as well as increased MDA level were observed in the WT TBI group compared to the WT sham group (*p* < 0.05, n = 6) (Fig. 1). No significant differences were observed between the WT and GCPII KO sham group. GCPII KO resulted in significantly diminished elevation of MDA level compared to WT TBI mice (*p* < 0.05, n = 6). In addition, GCPII deletion produced significant increases in GSH level, and SOD and GPx activities compared to their WT counterparts (all *p* < 0.05, n = 6).

### GCPII gene knockout reduced post-injury cell apoptosis in the injured cortex

**Fig. 2:**
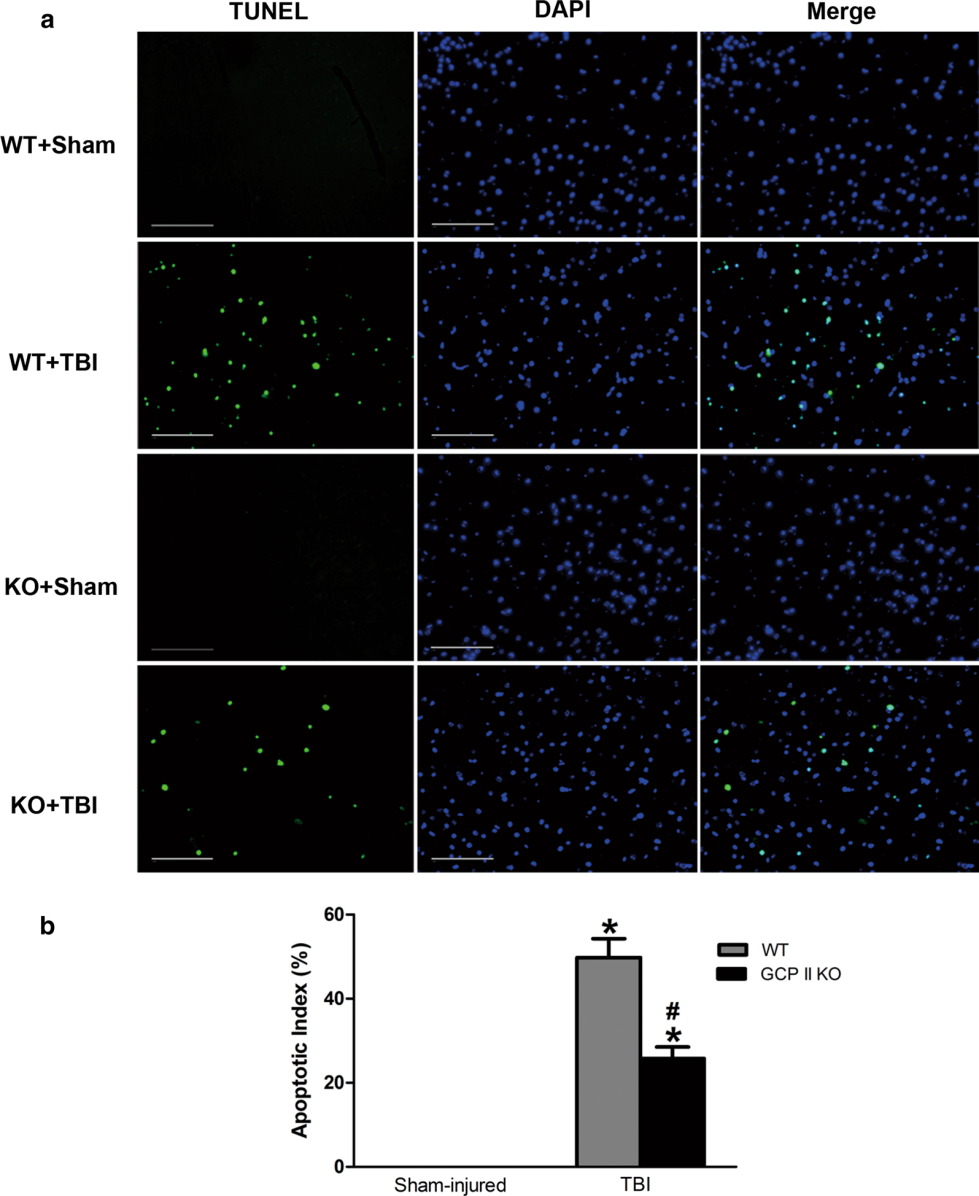
GCP II KO inhibited TBI-induced apoptosis. **a** Representative TUNEL-staining (*green*) and DAPI-stained (*blue*) brain sections of the penumbra area at 1.4 mm from the bregma (magnification ×200). The *scale bar* is 100 μm. **b** Quantification showed that GCP II KO markedly decreased the apoptotic index after TBI compared to the WT TBI mice. Data were represented as mean ± SEM (n = 6 per group); *p < 0.05, versus sham control of the same genotype; ^#^ *p* < 0.05, versus injured wild-type mice

TUNEL staining was performed to assess TBI-induced cell apoptosis. TUNEL-positive nuclei were not observed in both sham TBI groups (Fig. 2). The number of apoptotic cells in the contusion margin significantly increased in the TBI groups (both *p* < 0.05, n = 6). GCPII KO significantly diminished the number of TUNEL-positive nuclei in the cortex surrounding the injury core (*p* < 0.05, n = 6).

### GCPII KO stabilized the mitochondrial homeostasis and downregulated the expression of cleaved caspase-3

**Fig. 3:**
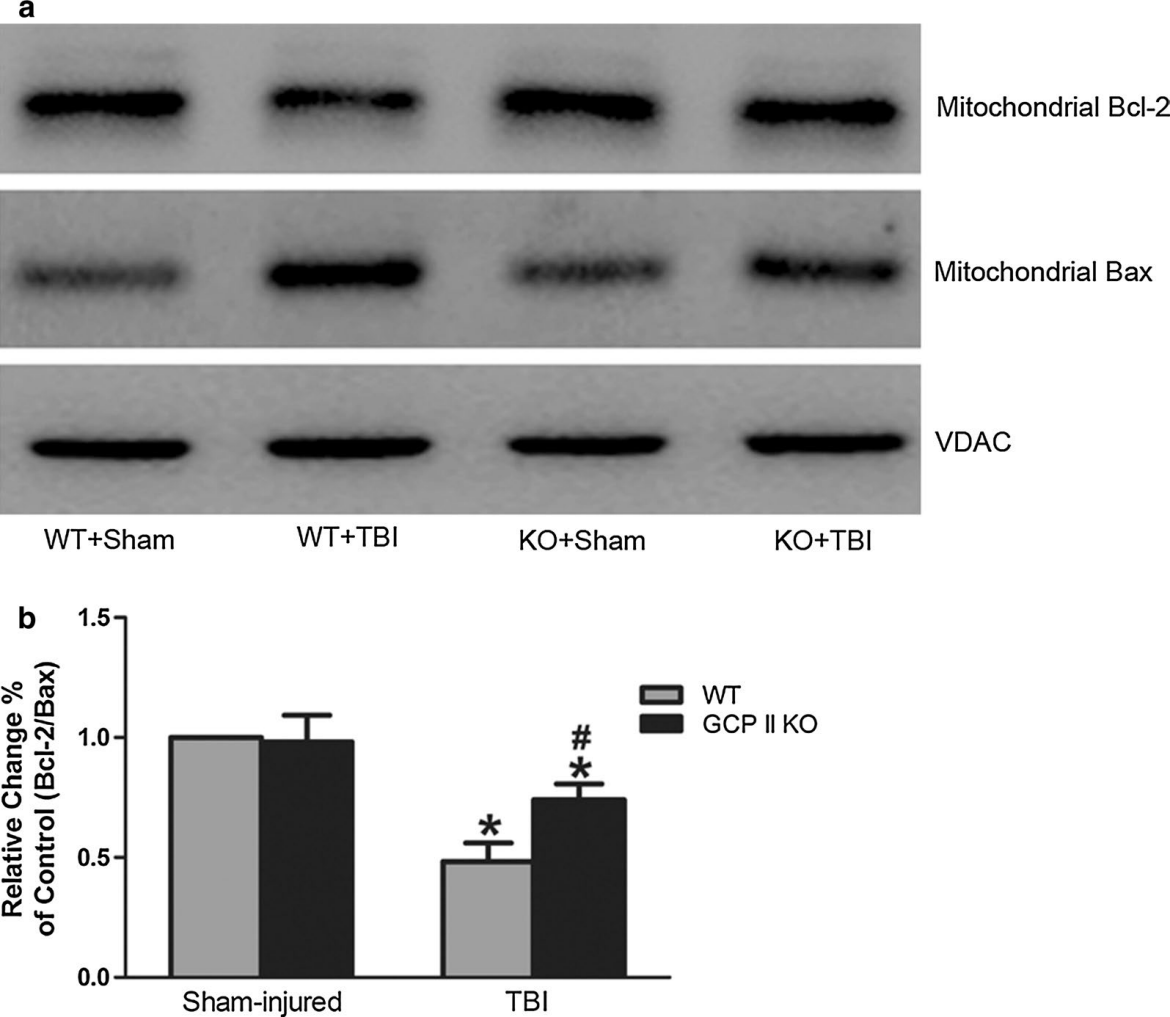
Effects of GCPII KO on the expression of mitochondrial Bcl-2 and Bax. Representative immunoblots (**a**) and densitometric analysis (**b**) revealed a significant reduction in the mitochondrial Bcl-2/Bax ratio following TBI in both wild type and KO mice. The ratio in GCPII KO mice was significantly higher than their WT counterparts. The immunoblot data were scanned and normalized to the density of VDAC. The ratio of the normalized data for the wild type/sham mice was given a value of one. Data from other groups are expressed as values relative to the value for the wild type/sham mice. Data were represented as mean ± SEM (n = 6 per group); *p < 0.05, versus sham control of the same genotype; ^#^ *p* < 0.05, versus injured wild-type mice

**Fig. 4:**
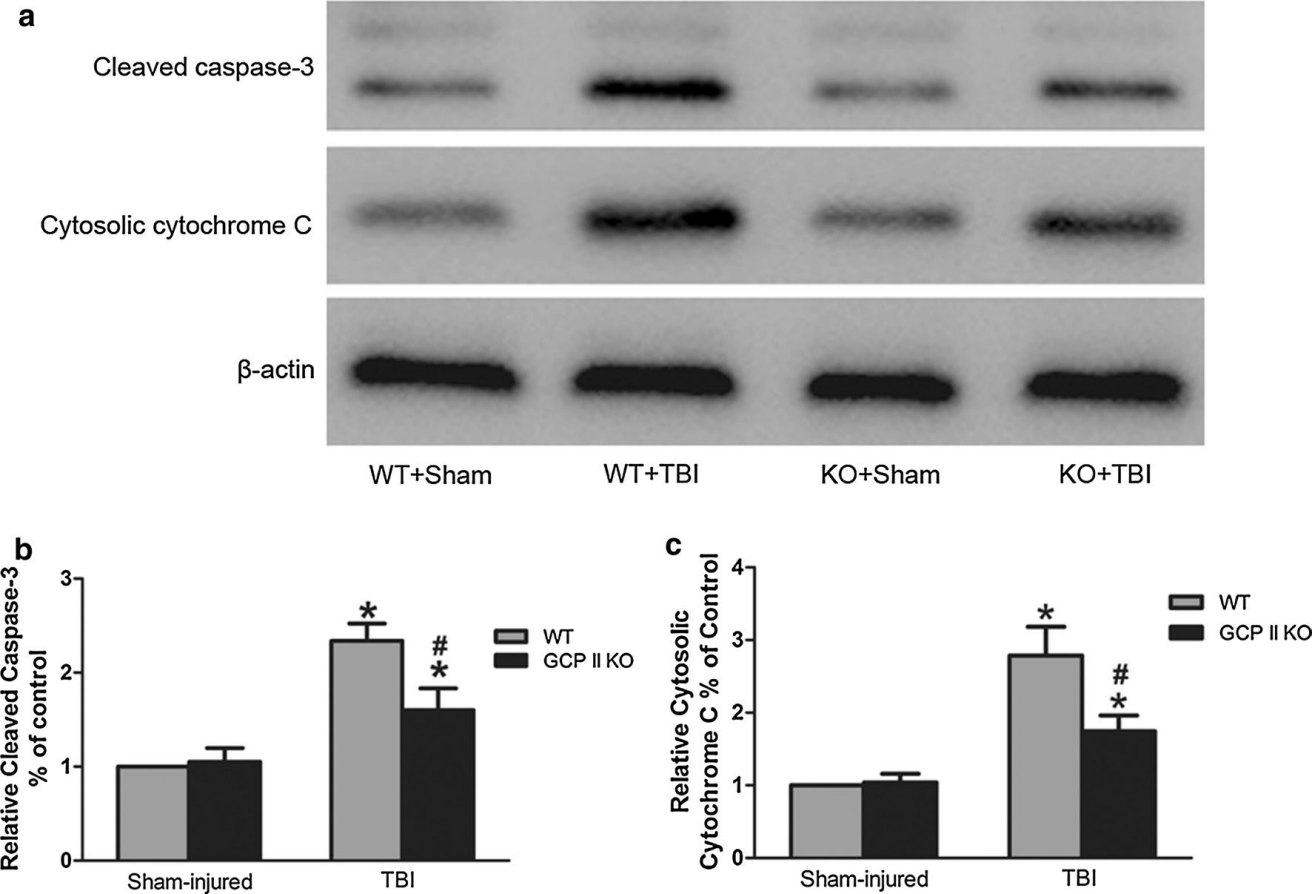
The expression of cleaved caspase-3 and cytosolic cytochrome c. **a** Representative immunoblots of cleaved caspase-3 and cytosolic cytochrome c in the ipsilateral cortex from sham and injured mice. Optical densities were normalized to beta-actin. The ratio of the normalized data for the wild type/sham mice was given a value of one. Data from other groups are expressed as values relative to the value for the wild type/sham mice. There was a significant increase in cleaved caspase-3 (**b**) and cytosolic cytochrome c (**c**) protein levels in wild type TBI mice and a significant moderation of this effect the GCPII KO mice. Data were represented as mean ± SEM (n = 6 per group); *p < 0.05, versus sham control of the same genotype; ^#^ *p* < 0.05, versus injured WT mice

A decrease in the ratio of mitochondrial Bcl-2 to Bax is proposed to reflect an increase in the permeability of the mitochondrial membrane and cell susceptibility to an apoptotic stimulus. There was increased Bax levels as well as reduced Bcl-2 levels in the mitochondrial fractions after TBI (Fig. 3a). The mitochondrial Bcl-2/Bax ratio in the WT TBI group was significantly lower than that in the WT sham group (*p* < 0.05, n = 6, Fig. 3b). GCPII KO significantly increased the ratio of Bcl-2/Bax in the mitochondria compared to their WT counterparts (*p* < 0.05, n = 6, Fig. 3b). As an apparent result of elevated mitochondrial permeability, the level of cytosolic cytochrome c significantly increased in the TBI groups (both *p* < 0.05, n = 6, Fig. 4a, c). However, the increase in the cytosolic cytochrome c level in the KO TBI mice was significantly less than that in the WT TBI mice (*p* < 0.05, n = 6, Fig. 4c).

Cleaved caspase-3 levels, a crucial apoptotic executor, were robustly upregulated in both groups after TBI (Fig. 4a, c), consistent with the result of TUNEL-staining. The GCPII KO group had significantly lower levels of cleaved caspase-3 compared to their WT counterparts (*p* < 0.05, n = 6).

## Discussion

Our previous study found that this strain of GCPII KO mice developed normally with no apparent differences compared with WT mice in terms of survival and standard neurological tests, but had lessened neuronal degeneration and astrocyte damage after TBI [17]. Data from the present study also indicate that the GCPII KO mice are less sensitive to TBI in terms of apoptosis in the penumbra around the impact site and additionally support the hypothesis that this protection is mediated by limiting oxidative stress.

It has long been recognized that head trauma induces an excessive release of glutamate and prolonged activation of Ca^2+^ ion channels [19]. Previous reports have demonstrated a close relationship between glutamate excitotoxicity and oxidative damage [20–23]. High levels of glutamate release and increased Ca^2+^ influx induces generation of reactive oxygen species and inhibition of GSH synthesis. Free radicals interact with unsaturated fatty acids in cell membranes to generate MDA, which serves as an index of lipid peroxidation [24]. Under normal conditions, the low levels of superoxide free radicals are scavenged by enzymatic or non-enzymatic antioxidants. However, a consequence of the overproduction of free radicals is depletion of GSH and reduction in the activity of CAT, GPx, and SOD, resulting in cellular redox disequilibrium. These observations support the proposal that reducing glutamate release helps to attenuate intracellular Ca^2+^ overload and oxidative stress.

Previous studies have reported that inhibition of GCPII exerts an endogenous protective role via increasing extracellular levels of NAAG, which selectively activates the mGluR3 and subsequently prevents further pre-synaptic glutamate release. For example, pretreatment with ZJ-43, an NAAG peptidase inhibitor, reduces the levels of glutamate, aspartate, and GABA following TBI, and protects neurons and astrocytes from undergoing neurodegenerative changes [15]. However, the exact cellular mechanism by which GCPII inhibition is protective against TBI has not been fully elucidated. In the current study, we focused on evaluating the injury-induced oxidative response using a GCPII KO model. In agreement with the theory that inhibiting glutamate exposure helps with GSH synthesis [3], the brain concentration of GSH in the KO mice was significantly higher than that of the WT mice following traumatic impact. Furthermore, the level of MDA was significantly reduced, and the activities of SOD and GPx were evidently enhanced due to GCPII KO. These data supports the hypothesis that the neuroprotective effects of GCPII KO can be attributed, at least in part, to limiting oxidative stress.

Mitochondria are important target organelles in the oxidative stress pathway and in affecting cell survival [25, 26]. The imbalance of mitochondrial Bcl-2 and Bax leads to an increased release of mitochondrial intermembrane space (IMS) proteins, such as cytochrome c, Smac/DIABLO, and apoptosis inducing factors (AIF) [27]. Those IMS proteins aid in the activation of caspase-3 and triggers apoptosis [5, 28]. In the present study, we observed decreased Bcl-2/Bax ratio, as well as increased cytochrome c leakage and cleaved caspase-3 expression in the tissues surrounding the primary injury. These data were consistent with the results of previous studies [18, 29]. Importantly, loss of GCPII reduced the number of TUNEL-positive neuclei and alleviated the alterations of these apoptosis-related proteins in the penumbra surrounding the primary impact area. These results support the hypothesis that GCPII KO better preserved the mitochondrial function and integrity, an action that likely contributes to decreased neurodegeneration following traumatic brain injury.

In addition to down-regulating presynaptic glutamate release, another possible neuroprotective mechanism of GCP II KO is also worth noting. Recent in vitro studies have shown that activating mGluR3 helps with recovering the endogenous GSH level [30], reducing ROS generation, increasing free radical scavenge and stabilizing mitochondrial function [31]. The exact mechanism by which mGluR3 regulates glial cell survival remains to be elucidated, but stimulation of MAP/ERK kinase and PI3 K/Akt signaling pathway may underlie protective effects of mGluR3 activation in these cells [32, 33].

## Conclusion

In conclusion, the current study confirmed that GCP II KO is neuroprotective against TBI induced oxidative damage and cell apoptosis and that effects correlate with the preservation of mitochondrial integrity. Further research will be required to determine the relationship among extracellular glutamate and NAAG levels and intracellular calcium in the GCP II KO mice after TBI in order to better prove that GCPII KO exerts its neuroprotective effects through the action of NAAG.

## Abbreviations

NAAG: *N*-acetylaspartylglutamate
GCPII: glutamate carboxypeptidase II
mGluR: metabotropic glutamate receptor
TBI: traumatic brain injury
CCI: controlled cortical impact
GSH: glutathione
SOD: superoxide dismutase
GPx: glutathione peroxidase
MDA: malondialdehyde
Ca^2+^: calcium
WT: wild-type
PBS: phosphate-buffered saline
PFA: paraformaldehyde
TUNEL: terminal deoxynucleotidyltransferase-mediated dUTP nick 30′-end labeling
TDT: terminal deoxynucleotidyltransferase
DAPI: 4′,6-diamidino-2-phenylindole
PVDF: polyvinylidene fluoride
PBST: PBS containing 0.05 % Tween 20
IMS: intermembrane space
AIF: apoptosis inducing factors
KO: knockout
